# A scoping literature review on the impacts of non-native species on the native terrestrial biodiversity of an oceanic island

**DOI:** 10.7717/peerj.20839

**Published:** 2026-02-25

**Authors:** Ghanishta Seeburrun, Cláudia Baider, Prishnee Bissessur, François Benjamin Vincent Florens

**Affiliations:** 1 Tropical Island Biodiversity, Ecology and Conservation Pole of Research, Department of Biosciences and Ocean Studies, Faculty of Science, University of Mauritius, Le Réduit, Mauritius; 2 The Mauritius Herbarium, Agricultural Services, Ministry of Agro-Industry, Food Security, Blue Economy and Fisheries, Le Réduit, Mauritius

**Keywords:** Biodiversity conservation, Biological invasion, Impact mechanism, Knowledge gaps, Mauritius, Non-native species, Scoping review

## Abstract

**Background:**

Many non-native species have been introduced to oceanic islands, with a subset becoming invasive, which pose the greatest threat to native terrestrial biodiversity. Yet, existing information on their impacts, particularly at the island scale, has rarely been synthesised. Addressing this gap is essential for revealing neglected aspects and for prioritising conservation management to optimise the use of scarce resources. Here, we use one volcanic oceanic island as a model to characterise the knowledge landscape about the impacts of non-native species on native biodiversity. Specifically, we (1) inventory the studied mechanisms through which non-native species affect native biodiversity; (2) evaluate the extent to which studies assess impacts at the level of biological organisation and/or biotic interactions; (3) assess the severity of impacts of non-native taxa and (4) highlight research gaps requiring most attention at the island scale.

**Method:**

Mauritius was used for a scoping review based on four databases (Dimensions, Google Scholar, ScienceDirect and SpringerLink) to systematically search and identify relevant studies on the impacts of non-native species on native terrestrial biodiversity. We used the Environmental Impact Classification for Alien Taxa (EICAT) framework and its extension, EICAT+ to categorise the impact mechanisms and magnitude of impact. We searched for all records from the inception of each database until November 2023. We included 62 publications in the final analysis, selected from a total of 1,760 articles screened following the PRISMA guidelines for scoping reviews.

**Results:**

We recorded a total of 273 impact records between non-native and native species. Non-native species of plants and mammals were the most common, affecting a wide range of native taxonomic groups. Negative impacts predominated (65.2%), followed by positive impacts (34.1%), with few neutral impacts (0.7%). Competition and predation were the most studied negative impact mechanisms, typically impacting many native species while positive impacts of non-native species were mainly associated with the provision of trophic resources and typically impacted few species. The main impacts of non-native species on native species studied were at the level of biological organisation (*N* = 169), while only 22 of the impact records investigated their effects on biotic interactions.

**Conclusion:**

Limited attention has been given to indirect mechanisms and the impacts of non-native species on biotic interactions. Future studies should prioritise these areas, as indirect mechanisms may reveal cumulative and often insidious pathways through which non-native species accelerate biodiversity loss, especially on islands. Investigating their effects on biotic interactions is critical for detecting cascading impacts to inform more effective and comprehensive conservation strategies.

## Introduction

Globalisation, driven by international trade and modern transportation, leads to the deliberate or accidental introduction of non-native species (terminology as per Soto et al., 2024) to new locations (Levine & D’Antonio, 2003; Hulme et al., 2008; Seebens et al., 2015; Bellard et al., 2016; Essl et al., 2018). Once established in their introduced range, some species become invasive as they spread and negatively impact the native biodiversity (Blackburn, Bellard & Ricciardi, 2019). Globally, at least 37,000 established non-native species are recorded, of which over 3,500 are classified as invasive (Roy et al., 2024; Soto et al., 2024). Consequently, invasive non-native species today counts among the leading threats to global biodiversity (Simberloff, 2013; Ricciardi et al., 2013; Pyšek et al., 2020), contributing to ∼16% of all documented extinctions (Roy et al., 2024), but also accounting for global socio-economic losses estimated at $423 billion annually (Diagne et al., 2021; Roy et al., 2024).

The impacts of non-native species are ecologically complex and context-dependent, varying according to the mechanism through which they act (Van Wilgen, Zengeya & Richardson, 2022; Carneiro et al., 2025). Invasive non-native species can negatively impact native biodiversity through mechanisms such as competition for resources (Ing & Mogren, 2020), predation (Pollock et al., 2019), transmission of diseases (Hatcher, Dick & Dunn, 2012), hybridisation (Elliott et al., 2010) and parasitism (IUCN, 2020; Pyšek et al., 2020). Collectively, they can reduce native species richness and abundance (Vilà & Weiner, 2004), cause native species’ extinction (Doherty et al., 2016), and even alter ecosystem functioning and services by changing hydrology, nutrient cycling, fire regimes, and other ecological processes (Dukes & Mooney, 2004; Miniat et al., 2021). Nonetheless, some non-native species have certain perceived benefits for native biodiversity in terms of productivity and ecosystem services (Goodenough, 2010). However, these are typically short-term, poorly quantified empirically and are often outweighed by their long-term negative impacts (Vilà et al., 2011). Many studies report the impact of non-native species without identifying the underlying mechanisms involved, or do so solely based on supposition. Clarifying these mechanisms can improve non-native species’ impact predictions and guide more targeted conservation strategies (Levine et al., 2003; White, Wilson & Clarke, 2006).

The impacts of non-native species on native species have been most commonly assessed through species-level responses, such as changes in abundance, survival, reproductive output (Vilà et al., 2011; Valiente-Banuet et al., 2015; Crystal-Ornelas & Lockwood, 2020; Heinen, Rahbek & Borregaard, 2020) and species extinctions (Rodda, Fritts & Chiszar, 1997). In contrast, comparatively fewer studies have examined the impact of non-native species on biotic interactions (Traveset & Richardson, 2014; Marciniak et al., 2024). Understanding the impact of non-native species on biotic interactions is essential, as the breakdown of mutualisms or trophic links can serve as precursor to biodiversity loss, often preceding population-level declines and species extinctions (Díaz et al., 2013). Recent records attribute 60% of global animal and plant extinctions at least in part to non-native species (Roy et al., 2024). About 75% of these are documented on terrestrial ecosystems, with 90% of global extinction events occurring on islands (Pyšek et al., 2020; Fernández-Palacios et al., 2021).

Oceanic islands are often referred to as biodiversity hotspots worldwide (Myers et al., 2000; Fernández-Palacios et al., 2021) since they host 20% of the world’s biota (Kier et al., 2009). The diversity of taxa on islands is often dictated by their geographic isolation and unique environmental dynamics, translating into relatively high levels of endemism (Kier et al., 2009). Such islands are generally characterised by fewer taxa usually with better abilities to naturally disperse and colonise new areas (Moser et al., 2018), and they often lack certain functional groups, like apex predators and grazing mammals (Mueller-Dombois, 1981; Keane, 2002). This makes insular biota more vulnerable to the impacts of invasive non-native species since they lack the required adaptive defence mechanisms and exhibit increased susceptibility to the diseases they vector (Paulay, 1994; Bellard et al., 2014). This is well illustrated by the accidental introduction of the brown tree snake to Guam, which drove ten native bird species to extinction because their limited defences rendered them more susceptible to predation (Savidge, 1987). Effective invasive non-native species management on oceanic islands is therefore essential, as these ecosystems face severe threats from invasions (Bellard et al., 2014) and have disproportionately contributed to global extinctions across multiple taxa (Caujapé-Castells et al., 2010; Fernández-Palacios et al., 2021).

Given that the impact of non-native species on biodiversity is context-dependent, efforts to address this threat on islands need to be sufficiently tailored to specific situations as closely as possible to be most efficient and effective (Crowley, Hinchliffe & McDonald, 2017). However, research is often done and conservation strategies are frequently implemented in a fragmented approach, without much of a holistic perspective about non-native species. This is largely because studies tend to be taxonomically focussed or consider a limited subset of mechanisms of non-native species impacts (Spooner, Smith & Sutherland, 2015; Christie et al., 2020; Christie et al., 2021). To remedy this situation, a synthesis at the level of an entire island is necessary, that considers the combined and overall impact of non-native and invasive non-native species (Sutherland et al., 2004; Dueñas et al., 2018). Such an approach allows for a more objective comparison of the various impacts of the different non-native species and processes and can produce valuable insights, such as identifying which non-native species or impact mechanisms are most severely acting and/or impacting the largest section of native biodiversity particularly in harmful ways. Despite its potential, comprehensive syntheses of this type have been rarely undertaken on islands (Haines et al., 2024).

The oceanic island of Mauritius provides an interesting setting to investigate the impacts of invasive non-native species, and may offer useful lessons applicable to islands in comparable ecological and economic contexts regarding their management. Since the first human colonisation of Mauritius in 1638, numerous non-native species have been introduced, both deliberately (for agriculture, biological control, hunting and pet trade) and accidentally (*e.g.*, horntail snails, rats) (Dulloo et al., 1996; Strahm, 1996; Dulloo, Kell & Jones, 2002; Mauremootoo, 2003; Griffiths & Florens, 2006; Cheke & Hume, 2008). Many of these non-native species have become invasive, leading to habitat loss, population declines and extinctions of native species (Safford, 1997a; Safford, 1997b; Cheke & Hume, 2008; Baider & Florens, 2011; Florens et al., 2017a; Florens et al., 2017b). Today, Mauritius is considered as one of the most ecologically devastated islands worldwide (Florens, 2013). Merely 4.4% of terrestrial habitat with high native content survive (Hammond et al., 2015), and what remains is both fragmented and heavily invaded by non-native species (Cheke & Hume, 2008; Florens et al., 2016; Florens et al., 2017b). Also, as a developing nation with a growing economy, Mauritius’ reliance on agriculture, tourism and trade is expected to remain high or rise, creating new pathways for non-native species introductions and heightening the risk of further invasions and spread (Seebens et al., 2018; Fernández-Palacios et al., 2021). All these characteristics position Mauritius as an interesting ‘laboratory’ for studying the dynamics of biological invasions in island ecosystems.

**Figure 1:**
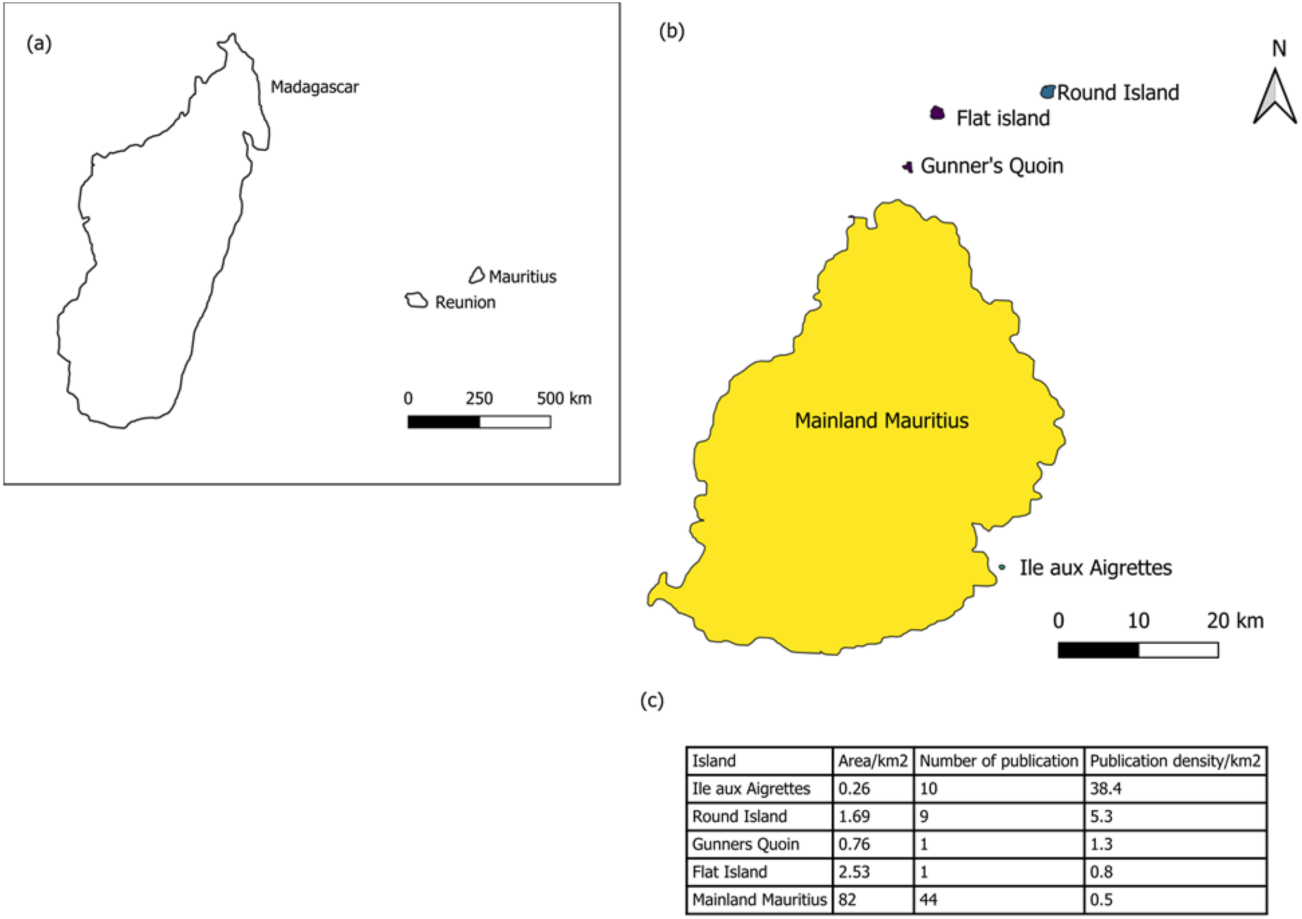
(A) Location of Mauritius in the southwest Indian Ocean shown relative to Madagascar and Réunion (inset), (B) Mainland Mauritius with its surrounding islets, (C) The number of publications per island. Colours in (B) represent publication counts grouped into three categories: purple indicates islands with one publication, green indicates islands with 9–10 publications, and yellow indicates mainland Mauritius with 44 publications; (C) represents area (km^2^) of each island, the number of publications retrieved, and publication density (publications per km^2^). For mainland Mauritius, the remaining 82 km^2^ of native habitat were used instead of the island’s total land area.

It is rare to find a synthesis that evaluates the scope and mechanisms by which non-native species impact the native terrestrial biodiversity, ecological processes and biotic interactions at a scale that can be used to support strategies or policies (Dueñas et al., 2018), and for the island of Mauritius, this remains a critical gap that this review addresses. The rationale is that existing knowledge is fragmented across taxa, habitats, and study designs, making it difficult to assess the extent to which research captures the full range of impacts and their relative importance. This underscores the need for a comprehensive synthesis to inform conservation priorities. A scoping review is ideal for this purpose because it maps existing evidence on a topic, summarises findings from studies with differing methods, and identifies major gaps that require further research (Tricco et al., 2016). This scoping review is intended for conservation managers, policy makers and scientists. By consolidating existing knowledge, it may highlight critical research gaps that require attention and provide a foundation for more informed conservation action and policy. Such insights may also resonate globally, extending their applicability beyond regional boundaries and informing strategies for biodiversity conservation and non-native species management in island systems worldwide since Mauritius’s experiences are used in textbooks of biodiversity conservation (Whittaker & Fernández-Palacios, 2007). Therefore, in this scoping review, we systematically searched and compiled past studies that show how non-native species impact Mauritius’s terrestrial biodiversity and its associated offshore islets (Fig. 1). We then (1) identified the mechanisms through which non-native species impact native biodiversity; (2) evaluated the extent to which studies have assessed the impact of non-native species at the level of biological organisation (individual, population, community) and/or on biotic interactions of native species; (3) assess the severity of impact of non-native taxonomic groups and (4) identified gaps in the current body of research to guide future ecological studies and conservation strategies.

## Methodology

### Literature selection

**Figure 2:**
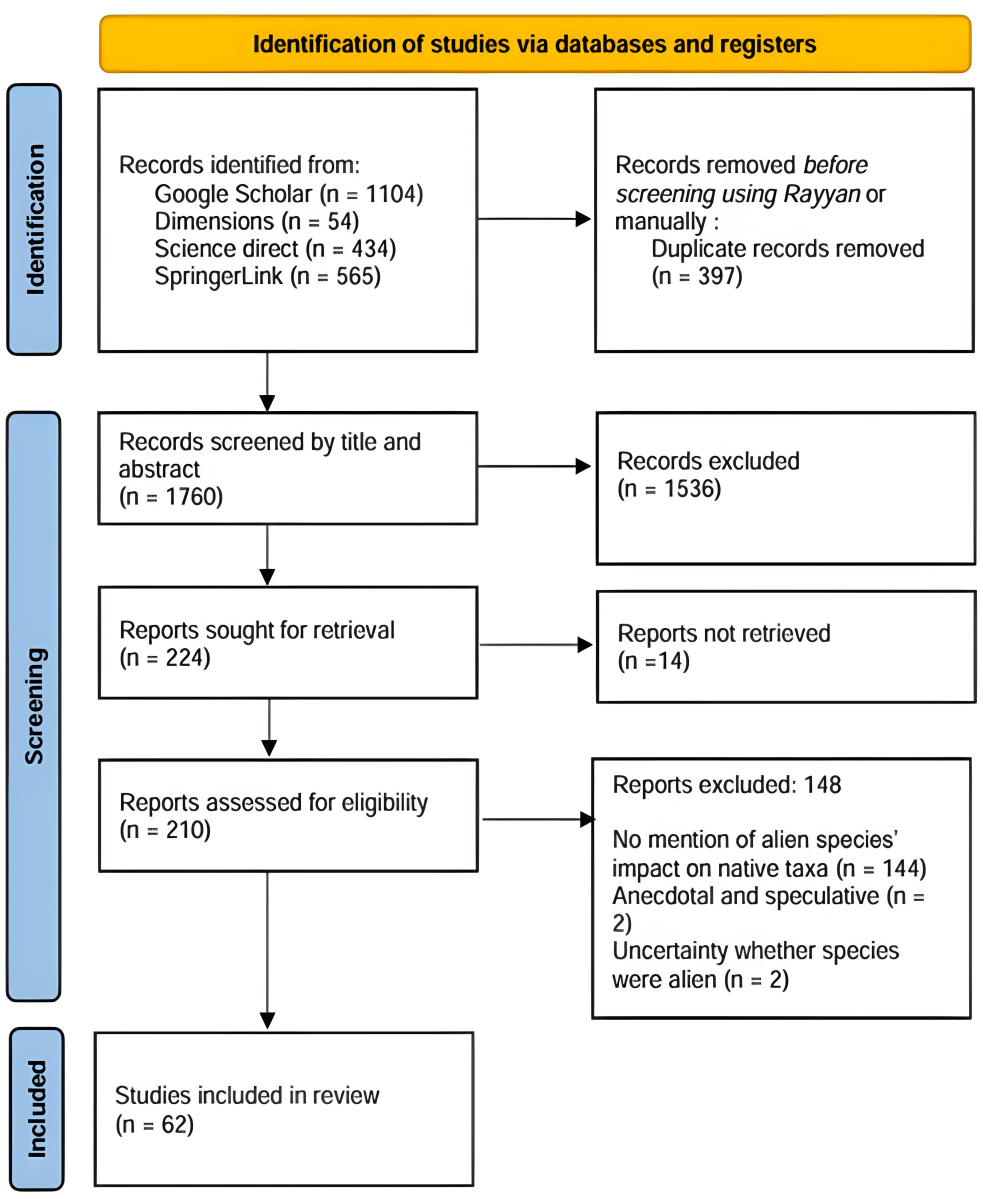
PRISMA diagram for the progression of papers included in this scoping review.

Our review protocol was designed following the PRISMA Extension for Scoping Reviews (PRISMA-ScR) (Tricco et al., 2018) (Fig. 2 for PRISMA flow diagram and Table S1 for PRISMA checklist). We systematically searched for published literature using four search engines (Dimensions, Google Scholar, ScienceDirect, and SpringerLink), as these provide broad coverage of relevant literature and are accessible either freely or through institutional subscriptions. The search spanned from the inception of each database up to November 2023. The search terms used were (“invasive alien species” OR “non-native species” OR “exotic species” OR “introduced species” OR “alien species”) AND “Mauritius”. To refine the search and exclude studies not relevant to the review’s scope, we applied exclusion filters using Boolean operators (*e.g.*, NOT) where supported (Atkinson & Cipriani, 2018; MacFarlane, Russell-Rose & Shokraneh, 2022). Terms related to agriculture, livestock, marine and human pathogens were excluded. The refined string, adapted as needed for each database’s syntax, included terms such as: NOT (“agriculture” OR “livestock” OR “marine” OR “human pathogen”). On a platform with limited Boolean functionality, like Google Scholar, exclusion terms were applied through the advanced search interface. The final search results were exported to Zotero, an open-source reference management tool. They were subsequently exported to the literature review management software Rayyan to facilitate the screening and selection process (Ouzzani et al., 2016).

 We established the following specific inclusion criteria to ensure the relevance and quality of the published evidence included in this review: (1) it documents an impact between non-native and native species; (2) it is based on original data or direct observations made by the author(s); (3) it explicitly describes the impacts grounded in empirical findings; and (4) it is written in one of the following languages: English, French, Portuguese, or Spanish. Studies were excluded if their information was anecdotal, speculative, or based on secondary sources. We also omitted review articles, conceptual papers, and publications without primary empirical data. Additionally, studies concentrating on agricultural crops, livestock, human pathogens, commercially cultivated tree species, marine or freshwater species, or medicinal plants were excluded.

We employed a two-stage screening process. First, we selected potentially relevant articles based on their titles and abstracts. Those that passed were then subjected to a more detailed full-text examination. Throughout both phases, two members of the research team (GS and CB) independently screened the articles. Before screening, 397 duplicates were automatically removed using Rayyan software or manually. In the first screening phase, both coders assessed all 1,760 abstracts from the search results. Substantial agreement between coders on accept/reject decisions was achieved (90.6% agreement, Cohen’s kappa = 0.68). Most disagreements stemmed from taxonomic uncertainties (*e.g.*, whether the species mentioned were non-native) or if the study was done in Mauritius or occasional coding errors. After discussion, disagreements remained for 53 records at the title-and-abstract stage, all of which proceeded to full-text screening. Combined with the 171 titles both reviewers initially agreed on, this resulted in 224 titles selected for full-text review, out of which 14 were inaccessible. To maintain consistency in applying full-text screening criteria, GS and CB independently reviewed the 210 full texts remaining after the initial title and abstract screening. After removing coding errors, only five texts showed disagreement (97.8% agreement, Cohen’s kappa = 0.94), all of which were re-evaluated. Any differences in determining article relevance at either screening stage were resolved through consensus among the coders. Ultimately, 62 scientific articles were included in the final analysis (Table S2).

### Data extraction

A data-charting form was prepared beforehand to identify which variables to extract. All data were then collected by the first author, verified by CB and PB, and jointly validated before analysis. Any disagreement regarding the relevance of the collected data was resolved through consensus among the authors. From the 62 articles that met the inclusion criteria, we identified all distinct impact records in which a non-native species was reported to have an impact on a native species. A single article could provide multiple records if it examined the impacts of several non-native species. When a study (*n*) assessed the effects of multiple non-native species on native species, these were recorded as separate cases (*N*). Additionally, if a non-native species was evaluated for impacts on multiple native species, each pair was documented individually. Consequently, each non-native/native species pair was considered a separate sample within the study. For each impact record, the following information was extracted: (i) the scientific names of the non-native and native species (where available), (ii) their respective taxonomic classes, *i.e.* Chordata (Amphibia (amphibians), Aves (birds), Mammalia (mammals), Reptilia (reptiles)), Arthropoda (Insecta (insects)), Mollusca (Gastropoda (molluscs)) and Plantae (Equisetopsida, Pinopsida, Polypodiopsida (ferns), Magnoliopsida (dicots), Liliopsida (monocots)). For Plantae results were grouped as ‘plants’ as none or a single impact were recorded for each Equisetopsida, Pinopsida and Polypodiopsida, and to be comparable with similar studies (Dueñas et al., 2018; Dueñas et al., 2021; Bacher et al., 2025; Carneiro et al., 2025), (iii) the type of study (experimental/observational), and (iv) the direction of the reported impact, categorised as positive, negative, or neutral based on the measured outcome of each impact record. In cases where the scientific names of either the non-native or native species were not specified, the species was recorded as ‘non-native’ or ‘native’, followed by the most specific taxonomic classification provided in the source.

### Classification of impact mechanisms

We then made a detailed assessment of each impact record by extracting data on the mechanism through which the non-native species impacted the native species. This was achieved by identifying the mechanism using the Environmental Impact Classification for Alien Taxa (EICAT) scheme for negative impacts (IUCN, 2020) and EICAT+ framework for positive impacts (Vimercati et al., 2022). EICAT has 12 standardised mechanisms, *i.e.,* competition, predation, hybridisation, transmission of disease, parasitism, poisoning/toxicity, biofouling/other direct physical disturbance, grazing/herbivory/browsing, chemical, physical and structural impact on ecosystems and indirect impacts through interactions with other taxa. EICAT+ has 10 mechanisms, *i.e.,* provision of trophic resources, overcompensation, hybridisation, disease reduction, dispersal facilitation, epibiosis/other direct provision of habitat, chemical, physical and structural impact on ecosystems and indirect impacts through interactions with other taxa. Each EICAT and EICAT+ impact mechanism was further classified as either ‘direct impact mechanism’ or ‘indirect impact mechanism’ based on whether the non-native species caused immediate effects on native species or operated through intermediate ecological processes, according to EICAT criteria (IUCN, 2020).

### Classification of impact types at the level of biological organisation and biotic interaction

Furthermore, for each impact record, we categorised the type of impact non-native taxa have on native taxa based on how the study authors described and evaluated the effect. We used the EICAT and EICAT+ frameworks (IUCN, 2020; Vimercati et al., 2022) with some modifications to assess these impacts. Since these frameworks classify impacts only by the level of biological organisation affected, namely: (1) individual impacts (changes in survival, growth, reproduction, defence, or immunocompetence); (2) population impacts (changes in population size); and (3) community impacts (changes in the area of occupancy), we added a category to cover impacts on biotic interactions. This additional category captures impact records where non-native taxa alter biotic interactions involving native species, such as pollination, seed dispersal, or herbivory, which EICAT/EICAT+ do not evaluate. Each impact record was assessed for evidence of effects in any of the four impact categories: (1) individual, (2) population, (3) community, and (4) biotic interaction. Since a single study might report impacts in multiple categories, impact records could be categorised accordingly. Only those impact records reporting at least one measurable outcome in any of these categories were considered for impact classification. This classification enabled us to evaluate the extent to which studies focus on the impacts of non-native species on the level of biological organisation of native species *versus* their impacts on biotic interactions, thereby allowing us to identify any gaps that may exist in the available literature.

### Severity of impacts of non-native species at the level of taxonomic groups

For each impact record that had negative and positive effects, we evaluated the severity of the non-native species’ impact on native taxa using one of the five impact-magnitude levels defined in the EICAT and EICAT+ frameworks (IUCN, 2020; Vimercati et al., 2022). For negative impacts, we assigned one of the following EICAT impact magnitude, based on the highest level of biological organisation for which evidence was reported: (1) Minimal Concern (MC)—negligible decrease in native individual performance; (2) Minor Impact (MN)—decrease in native individual performance but no decrease in population size; (3) Moderate Impact (MO)—decrease in native population size; (4) Major Impact (MR)—reversible decrease at the community level through local or sub-population extinction (or presumed extinction); and (5) Massive Impact (MV)—irreversible decrease at the community level through the local or global extinction (or presumed extinction). For positive impacts, we applied the corresponding EICAT+ impact magnitude: (1) Minimal positive impact (ML+)—negligible increase in native individual performance; (2) Minor positive impact (MN+)—increase in native individual performance but no increase in native population size; (3) Moderate positive impact (MO+)—increase in population size; (4) Major positive impact (MR+)—transient increase in native community through local re-establishment or prevention of extinction; and (5) Massive positive impact (MV+)—long-term increase in native community through local re-establishment or prevention of extinction. The impact magnitude of each impact record was assessed only for impacts reported at the level of biological organisation levels recognised by EICAT. Impact records that did not report any measurable impact outcome were classified as Data Deficient (DD), in accordance with EICAT guidelines, as the available information was insufficient to determine the presence or severity of impacts.

### Data analysis

To ensure that agreement between the two coders during the screening stage was not due to chance, we calculated Cohen’s kappa statistic and used 0.6 as the minimum acceptable threshold for inter-rater reliability (Cohen, 1960; Landis & Koch, 1977; Mologni, Moffat & Pither, 2023). Descriptive statistics (counts and percentages) were used to summarise publication characteristics, impact directions, impact mechanisms, impact types, impact magnitudes, and the taxonomic identity of native and non-native species. Data visualisation, including Sankey diagrams, dot plots, UpSet plots, and cumulative curves were done in R version 4.3.2 (R Core Team, 2023), and the spatial heat maps were generated in QGIS version 3.40.10.

## Results

### General search results

In all, 62 search results from 1977 to 2023, met all the inclusion criteria for the final analysis including 58 peer-reviewed journal articles, two conference proceedings, one book chapter, and one journal supplement article. The cumulative number of publications increased steadily from 1977 to 2023, with a marked acceleration after the early 2000s (Fig. S1). Publication density was highest for offshore islets, with Ile aux Aigrettes (10 publications; 38.4 per km^2^), Round Island (nine publications; 5.3 per km^2^), Gunner’s Quoin (one publication; 1.3 per km^2^) and Flat Island (one publication; 0.8 per km^2^) (Fig. 1). In contrast, mainland Mauritius recorded 44 publications (0.5 per km^2^ across the remaining 82 km^2^ of native habitats) (Fig. 1, Table S3).

The review identified 273 impact records involving non-native and native species (See Table S4 for impact records), as several publications reported more than one instance of non-native impacting native species. Most of these were observational (90.1%, *N* = 246), and 9.9% (*N* = 27) were experimental. Among the observational studies, most were quantitative studies based on detailed field data (90.2%, *N* = 222), while the remainder were descriptive field studies (9.6%, *N* = 24). Based on geographic origin, 137 species were identified as distinct native species and 94 as distinct non-native species (see Table S5). The studied impacts of non-native species varied across native taxonomic groups. Most affected native taxa were plants (87 species), followed by invertebrates (32 species, comprising 19 molluscs and 13 insects), reptiles (11 species), birds (six species) and mammals (one species).

### Impact mechanisms through which non-native species impact native species

**Figure 3:**
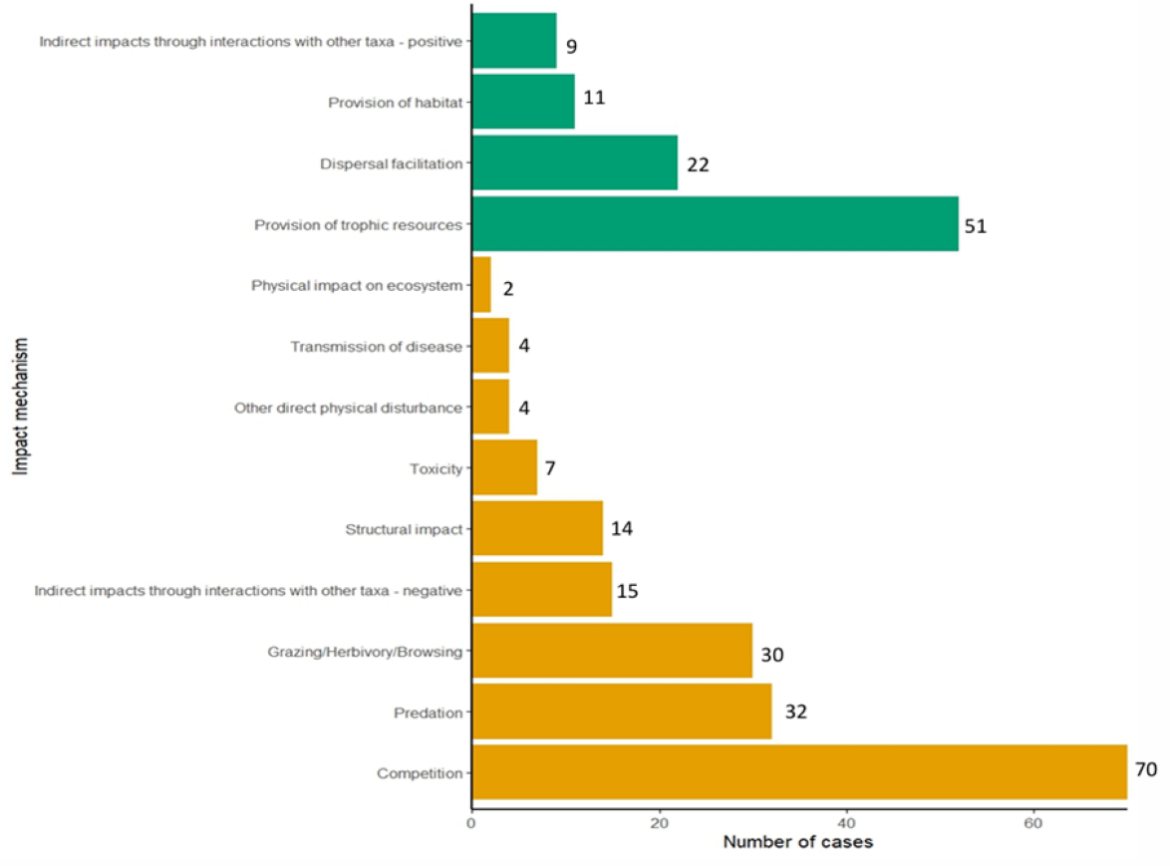
Counts of recorded impact mechanisms through which non-native species affected native species in Mauritius, separated by direction of impact (negative is orange, positive is green). Bars represent the number of impact records attributed to each mechanism.

Among the 273 impact records, 65.2% (*N* = 178) were of negative impacts, 34.1% were of positive impacts (*N* = 93) and 0.7% (*N* = 2) were of no impact. Overall, 90.8% of impact mechanisms were direct (*N* = 248), while indirect mechanisms were rare, comprising only 9.2% (*N* = 25). We identified nine EICAT impact mechanisms and four EICAT+ mechanisms, highlighting the diverse pathways through which non-native species impact native biodiversity (Fig. 3). Within negative impacts, competition was most frequent (39.3%, *N* = 70), followed by predation (18.0%, *N* = 32) and grazing/herbivory/browsing (16.9%, *N* = 30). Less common mechanisms included indirect interactions with other taxa (8.4%, *N* = 15), structural impacts (7.9%, *N* = 14), toxicity (3.9%, *N* = 7), direct physical disturbance (2.2%, *N* = 4), disease transmission (2.2%, *N* = 4), and ecosystem-level physical impacts (1.1%, *N* = 2). Within positive impacts, provision of trophic resources dominated (54.8%, *N* = 51), followed by dispersal facilitation (23.7%, *N* = 22), habitat provision (11.8%, *N* = 11), and indirect interactions (9.7%, *N* = 9).

**Figure 4:**
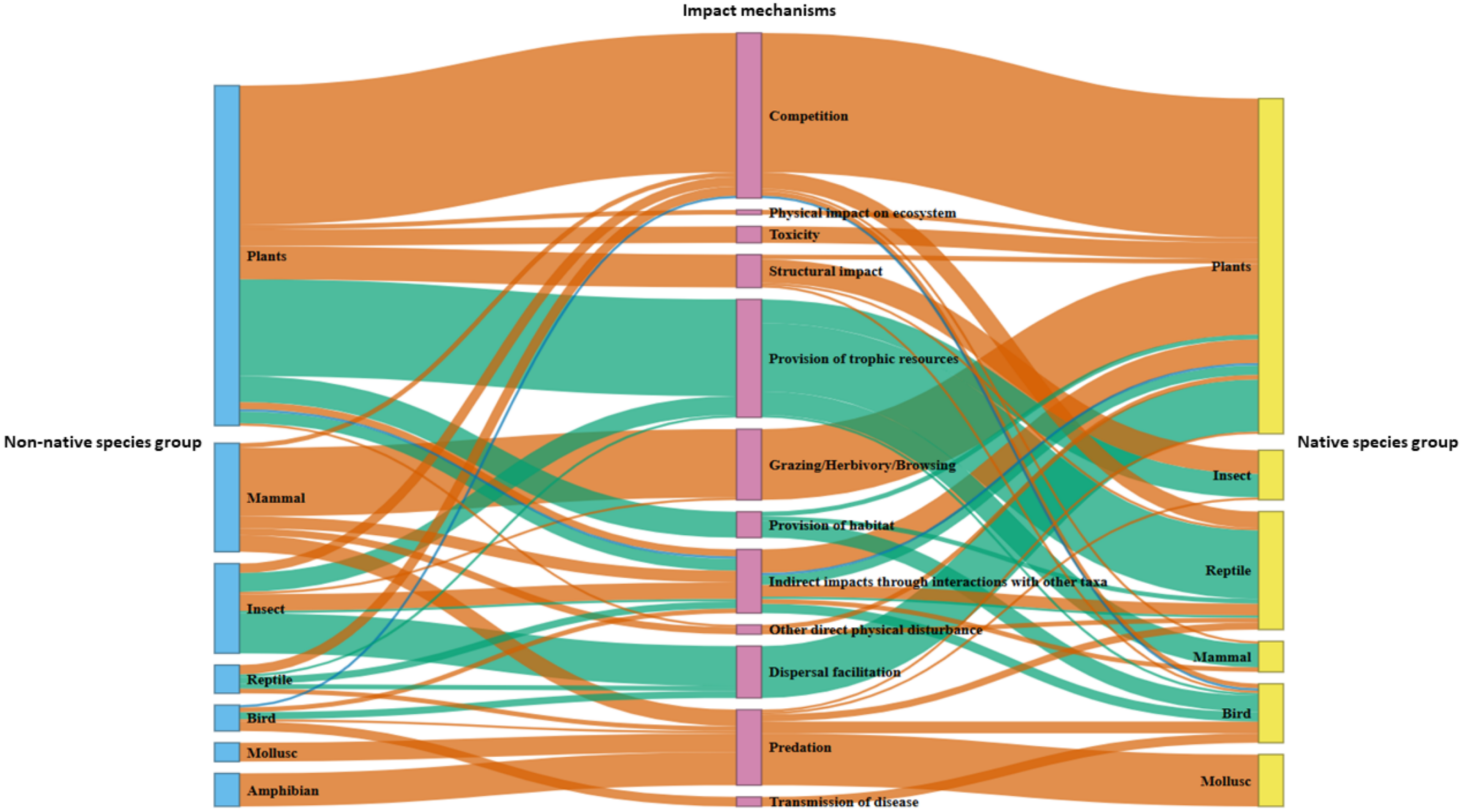
Sankey diagram illustrating ecological interactions between non-native and native taxa across 273 impact records. Flows represent the direction and frequency of impacts, connecting non-native taxonomic groups (left), impact mechanisms (centre), and affected native taxonomic groups (right). The width of nodes and flows is proportional to the number of reported interactions. Node size indicates interaction frequency, not taxonomic richness—thus, a single non-native species may appear larger if it caused multiple impacts. Flow colours represent the direction of impact: negative (orange), positive (green), and neutral (blue).

The reviewed studies show that non-native plants and mammals exert a profound ecological impact by interacting through multiple mechanisms and affecting diverse native taxa (Fig. 4). Non-native plants pose significant threat to native plants, mainly through competition for limiting resources (*N* = 59) (*e.g.*, Parnell et al., 1989; Baider & Florens, 2006; Baider & Florens, 2011; Baider & Florens, 2013; Virah-Sawmy et al., 2009; Monty, Florens & Baider, 2013; Florens et al., 2016; Krivek et al., 2020; Bissessur et al., 2023) and through the release of toxins into the soil (*N* = 7) (Sujeeun & Thomas, 2022). They also adversely impact native insects (*N* = 10), birds (*N* = 1) and reptiles (*N* = 1) by altering habitat structure (Kaiser-Bunbury, Memmott & Müller, 2009; Florens et al., 2010). Also, while non-native plants can occasionally provide resources for certain taxa, including reptiles (*N* = 21) (Bullock, 1977; Olesen, Eskildsen & Venkatasamy, 2002; Pernetta, Bell & Jones, 2005; Zuël et al., 2012; Tercel et al., 2022), mammals (*N* = 10) (Nyhagen et al., 2005), and insects (*N* = 10) (Olesen, Eskildsen & Venkatasamy, 2002), their overall impact remains largely detrimental.

Non-native mammals also exert strong negative impacts, mainly trophic, through herbivory and predation. Rabbits (*Oryctolagus cuniculus*) and goats (*Capra hircus*), now extirpated from the islets (Bullock, 1977; Bullock & North, 1984; North & Bullock, 1986; North, Bullock & Dulloo, 1994), suppressed regeneration by grazing on any accessible plant species. Rabbits mainly grazed on seedlings and herbaceous plants, while goats additionally also browsed shrubs and stunted trees (*N* = 10). Collectively, these mammals caused overgrazing, which led to soil erosion and a decline in soil fertility, triggering a series of degradations that increased plant mortality. This, in turn, may have contributed to the extirpation of many plant species. Macaques (*Macaca fascicularis*) (*N* = 17) and black rats (*Rattus rattus*) (*N* = 2) reduce the reproductive success of native plants by consuming their reproductive structures like flowers (Bissessur et al., 2019) and unripe fruits which leads to premature seed death (Baider & Florens, 2006). In addition, macaques, rats and mongooses (*Herpestes javanicus*) prey on native birds (*N* = 5) (Safford, 1997b; Carter & Bright, 2002; Roy, Jones & Harris, 2002), while rats also prey on molluscs (*N* = 1) (Florens, Daby & Jones, 1998) and seeds of native plants (*N* = 1) (Baider & Florens, 2013).

### Patterns and distribution of studies assessing non-native species impacts on native species at the level of biological organisation and biotic interactions

**Figure 5:**
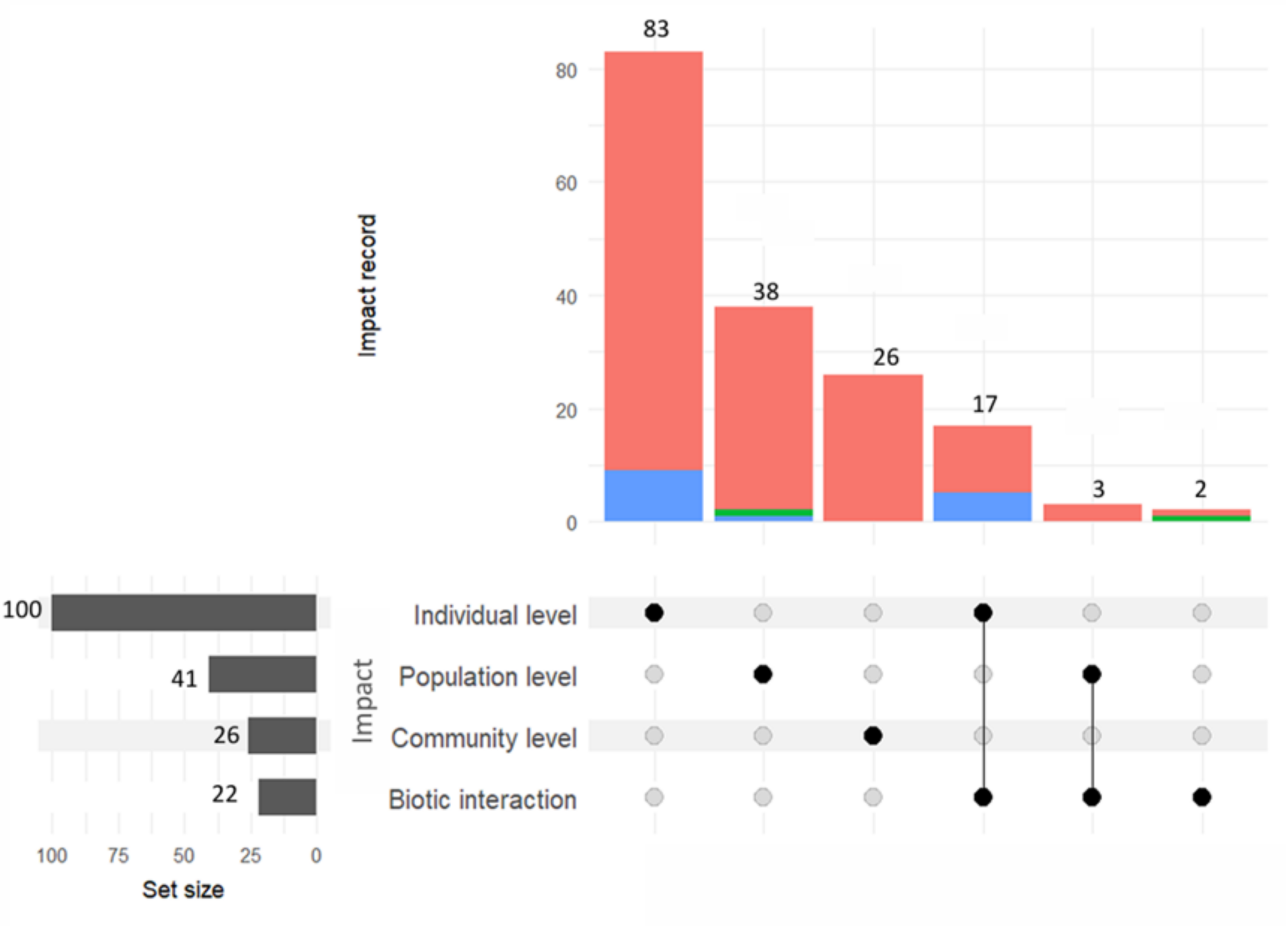
Number of impact records and the effects of non-native species across different impact categories. Vertical bars (intersection sizes) show the number of impact records assigned to each combination of categories, while horizontal bars (set sizes) indicate the total number of records within each individual category. Filled nodes and connecting lines represent the categories included in each intersection. Impacts were classified into four categories: (1) Individual—effects on native individual performance (*e.g.*, survival, growth, reproduction); (2) Population—changes in native population size or density; (3) Community—changes in the area of occupancy; and (4) Biotic interaction—non-native species altering ecological interactions involving at least one native species. Bar colours denote whether impacts were reported as negative (salmon), positive (blue), or neutral (green).

Our review revealed that across all impact records (*N* = 273), only 62% (*N* = 169) assessed the impact of non-native species at the level of biological organisation (individual, population, or community) or on biotic interaction. The remaining 38% (*N* = 104) did not report any detectable impact on native species at any of the impact categories (individual, population, community, biotic interaction). Most impact records (88.2%, *N* = 149) assessed the impact of non-native species within a single impact category. Among these, the majority focused on the individual level (*N* = 83), followed by the population (*N* = 38) and community levels (*N* = 26), with only two records assessing impacts solely on biotic interactions (Fig. 5; See Table S6). The remaining 11.8% (*N* = 20) of impact records assessed impacts across two impact categories. Nearly all of these multi-category assessments involved biotic interactions, most frequently in combination with individual-level impacts (*N* = 17) and, to a lesser extent, with population-level impacts (*N* = 3). Overall, impacts on biotic interactions were the least studied (*N* = 22), compared with those assessed at levels of biological organisation (*N* = 167). Among the impact records that evaluated the impact of non-native species on biotic interactions, the most commonly assessed was pollination (*N* = 11) (Hansen, Olesen & Jones, 2002; Kaiser, Hansen & Müller, 2008; Kaiser-Bunbury, Memmott & Müller, 2009; Hansen & Müller, 2009; Kaiser-Bunbury, Memmott & Müller, 2009), followed by commensal relationships involving native reptiles using vegetation for shelter (*N* = 4) (North, Bullock & Dulloo, 1994), and prey–predator interactions (*N* = 4) (Carter & Bright, 2002; Carpouron et al., 2023). Fewer studies have tested the impacts on frugivory (*N* = 1) (Krivek et al., 2020), seed dispersal (*N* = 1) (Hansen & Müller, 2009), and herbivory (*N* = 1) (Lach, Tillberg & Suarez, 2010).

### Impact severity of non-native species

**Figure 6:**
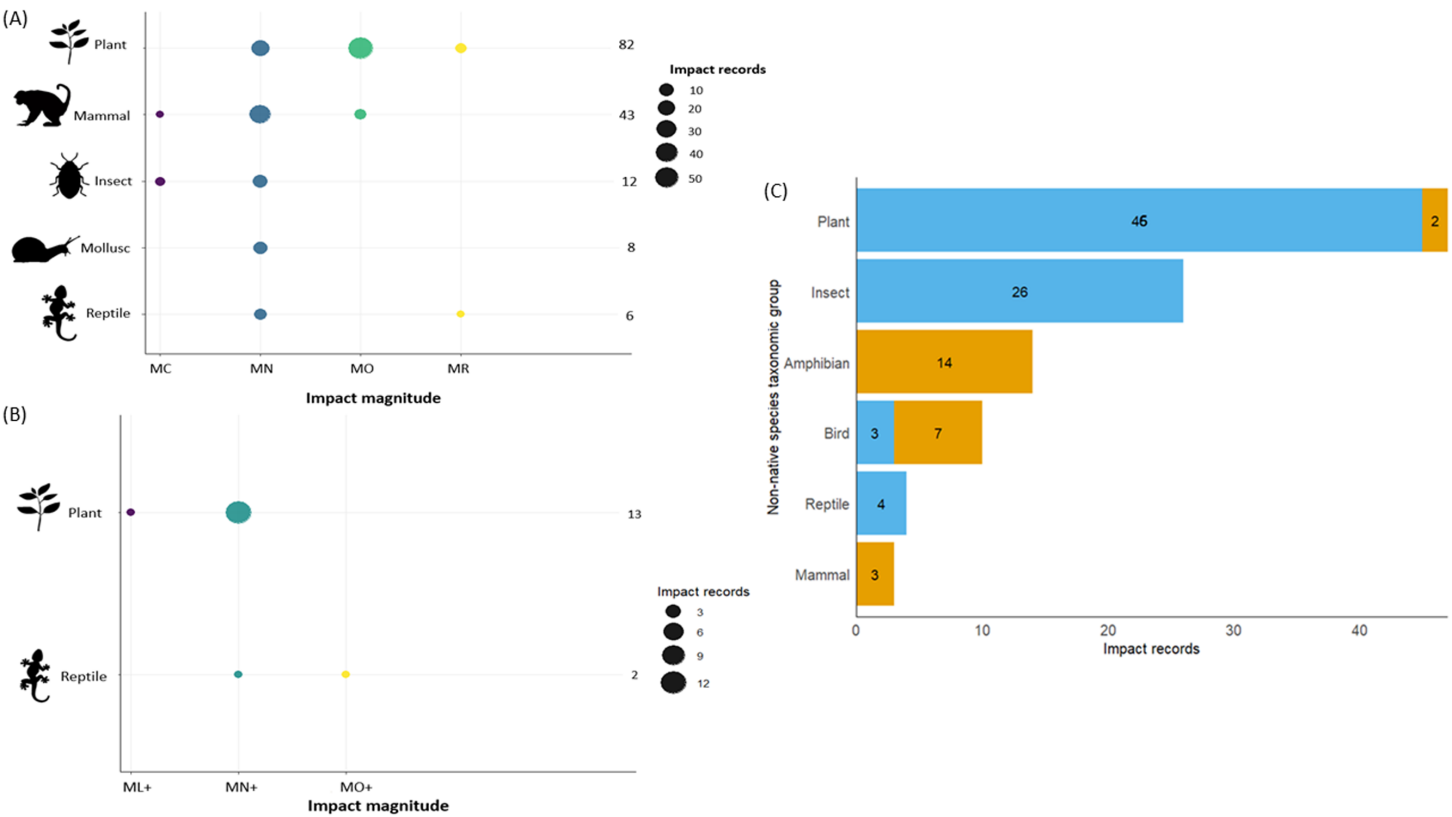
Impact magnitudes of non-native species across taxonomic groups. (A) Negative impacts and (B) positive impacts of non-native species on native taxa, classified according to the EICAT/EICAT+ magnitude categories. Negative impact magnitudes include Minimal Concern (MC), Minor (MN), Moderate (MO), Major (MR), and Massive (MV). Positive impact magnitudes include Minimal positive (ML+), Minor positive (MN+), Moderate positive (MO+), Major positive (MR+), and Massive positive (MV+). For each taxonomic group, the number shown on the *y*-axis indicates the total number of impact records. Circle size represents the number of impact records within each magnitude category, and colours (MC - purple, MN - blue, MO - green, MR - yellow, ML+ - purple, MN+ - green and MO+ - yellow) distinguish magnitude levels. (C) Taxonomic groups for which impact magnitude could not be assigned and were classified as Data Deficient (DD). Bars show the number of negative (orange) and positive (blue) impact records lacking sufficient information for magnitude assignment. Numbers within bars represent the count of DD impact records for each impact type.

Out of 273 impact records, 166 could be assigned an impact magnitude, while 105 were classified as Data Deficient (DD) due to insufficient information (Fig. 6; See Table S6). Two records indicated neutral effects and were not given a magnitude. Among the 151 negative impact records, impact magnitudes ranged from MC to MR. Non-native plants had the most harmful impact on native species, mainly by decreasing native population sizes (MO = 57) and, in a few cases, causing local extinctions (MR = 3) (Fig. 6A; See Table S6). They were followed by non-native mammals (MO = 43) and reptiles (MR = 1). Conversely, for positive impact records (*N* = 15), only non-native plants (*N* = 13) and reptiles (*N* = 2) received impact magnitudes (Fig. 6B; See Table S6). Positive impacts by non-native plants mostly improved native species’ performance (MN+ = 12), while reptiles increased both native individuals’ performance (MN+ = 1) and population size (MO+ = 1).

## Discussion

This scoping review identified 273 cases in which non-native species affected native species through various mechanisms, with most impacts being negative, though some positive impacts were documented. The most frequently reported mechanisms by which non-native species affected native species were direct. Negative impacts were primarily caused by competition, predation, and herbivory, while positive effects resulted from the provision of trophic resources, pollination, seed dispersal, and provision of habitat. We also found that non-native plants and mammals predominated in the reviewed impact records and were responsible for most of the harmful effects on native species. Moreover, most impact records examined the impact of non-native species on native species based on the level of biological organisation they affected (individuals, populations, and communities), while only a few investigated their impact on biotic interactions.

### Biological implications

Most reported impact mechanisms of non-native species in Mauritius were direct, while indirect impact mechanisms were much less frequently documented. This situation likely stems from the prevailing focus on pairwise biotic interactions, *i.e.,* studies that investigate direct impact mechanisms (*e.g.*, competition, predation, herbivory, provision of trophic resources) of individual non-native species on specific native species (Goodenough, 2010; Schlaepfer, Sax & Olden, 2011; Dueñas et al., 2018; Tamburello & Litt, 2023). Direct impact mechanisms are easier to observe because they involve frequent, visible biotic interactions between non-native and native species, whereas indirect mechanisms (*e.g.*, apparent competition, disruption of mutualisms, facilitation) are more complex and harder to quantify, and thus are less often studied in invasion science (Levine & D’Antonio, 2003; White, Wilson & Clarke, 2006; Carneiro et al., 2025). However, neglecting the impact of indirect mechanisms, especially biotic ones, can lead to an underestimation or overestimation of the threat posed by a given non-native species, potentially weakening the effectiveness of conservation policy. Through such mechanisms, a non-native species can have cascading effects across trophic levels and ecological networks, and either amplify or diminish the impact of other factors (White, Wilson & Clarke, 2006; Zarnetske et al., 2013; Allen et al., 2020). In natural ecosystems, non-native species rarely act in isolation; their co-occurrence can lead to cumulative effects that may be additive, synergistic or antagonistic (Genes & Dirzo, 2022). Consequently, such indirect impacts can often lead to more significant and rapid biodiversity loss, effects that are frequently overlooked in single-species studies (Simberloff, 2006; Linders et al., 2019; Sesay et al., 2024).

Non-native species can have biotic indirect impacts on native species by facilitating or suppressing interactions between a mediator species (M) and a native species of interest. In this context, we refer to mediator species (non-native or native) as those whose interactions with a native species are modified by the presence of a non-native species (Strauss, 1991; Wootton, 2002; White, Wilson & Clarke, 2006). For example, invasive non-native plants (M) facilitate the infestation of invasive non-native ants in the flowers of *Roussea simplex* Sm. (Bissessur, Baider & Florens, 2020), an endemic endangered plant, which in turn disrupt their pollination by endemic geckos (Hansen & Müller, 2009). In some cases, non-native species may facilitate the positive impact of native mediator species. For example, within invaded forests where native palms have gone extinct, traveller’s palms (*Ravenala madagascariensis* Sonn.), facilitate predation on endemic geckos (M) by the endemic Mauritius kestrel (*Falco punctatus*), potentially by increasing gecko density or detectability, and this boosts the bird’s breeding success (Carpouron et al., 2023). Non-native species can also suppress negative impacts of non-native mediator species. For example, the endemic Mauritius Fody (*Foudia rubra*) faces reduced nest predation by the invasive non-native macaque (*M. fascicularis*) (M) when nesting in non-native trees like Japanese cedar (*Cryptomeria japonica* (L.f.) D.Don) and swamp mahogany (*Eucalyptus robusta* Sm.) (Carter & Bright, 2002). These examples illustrate that in a transformed ecosystem, some non-native species can bring both harmful and beneficial indirect impacts by altering interactions between mediator species and native taxa.

Another important insight from this review is the imbalance in research focus. Most impact records focused on how non-native species affect native taxa by decreasing individual performance and causing population declines, while giving little attention to their impact on biotic interactions, as also seen elsewhere (Valiente-Banuet et al., 2015; Kollmann et al., 2016). Emphasising impacts based on the level of biological organisation affected might underestimate the broader ecological impact of non-native species on biotic interactions like pollination, seed dispersal, decomposition, and nutrient cycling. Disruptions to these biotic interactions can even have cascading effect on the structural and functional integrity of entire ecosystems (Didham et al., 2005; Traveset & Richardson, 2006; Traveset & Richardson, 2014; Mortensen, Dupont & Olesen, 2008; Cadotte, Carscadden & Mirotchnick, 2011; Rogers et al., 2017). The paucity of such studies potentially emanates from the difficulty in detecting and measuring these biotic interactions, which tend to be scale and time dependent (*e.g.*, seasonality, phenology) and often require detailed behavioural observations, network-based analyses, and controlled experiments (Heinen, Rahbek & Borregaard, 2020; Marciniak et al., 2024). These approaches are therefore more time-consuming and resource-intensive, a challenge exacerbated in Mauritius where research and development expenditure is 0.3% of gross domestic product (GDP) compared to an average of 2.1% in comparable upper-middle-income countries (Schneegans, Straza & Lewis, 2021; WIPO, 2024; Jarvis et al., 2025). To overcome this hurdle, more reliable and long-term financial resources, time and technical expertise need to be allocated to such studies to expand our knowledge on these areas. This can be facilitated by fostering regional and international collaborations, for example, through coordinated distributed experiments (Fraser et al., 2013; Borer et al., 2014) and global biodiversity monitoring networks (Guerrero-Ramírez et al., 2025), adopting low-cost approaches like citizen science, automated monitoring systems and making more efficient use of limited funding by prioritising understudied topics, including biotic interactions (Hill et al., 2018; Gouraguine et al., 2019; Wiedenfeld et al., 2021; Johnson et al., 2022; Dimson et al., 2023; Chalmers et al., 2023). Some of these studies could be standardised and applied to other islands to improve detection (or not) of invasion patterns.

### Management implications

Management responses tend to address the most well-documented pressures, particularly competition and predation by non-native species. However, focusing primarily on direct impacts can lead to ineffective strategies, particularly when non-native species interact with other species, potentially leading to synergistic or antagonistic effects on native biodiversity (Genes & Dirzo, 2022). Therefore, conservation managers should take into consideration both direct and indirect mechanisms by which non-native species impact native species when designing evidence-based interventions, to ensure they target the underlying drivers of impact and avoid cascading effects or unintended consequences.

Given that most of the studies assessed the impact of non-native species on native species based on the level of biological organisation it affects, such as their growth, abundance, survival, or reproduction, the existing knowledge risks inducing conservation managers to adopt more of a species-centric approach, focusing mainly on maintaining viable populations of individual species through management tools like captive breeding (Simberloff, 1998; Florens, 2013). However, a self-sustaining population of species mainly occurs when the functional interaction network among those species is restored, rather than simply achieving an increase in abundance (Colwell, Dunn & Harris, 2012; Florens, 2013; McConkey & O’Farrill, 2015; Valiente-Banuet et al., 2015; Heinen, Rahbek & Borregaard, 2020). Therefore, it is vital for conservation managers to adopt more holistic, interaction-focused management strategies to restore and sustain functional interactions among species for enhanced ecosystem resilience (Heinen, Rahbek & Borregaard, 2020; Dansereau et al., 2025; Baguette, Baider & Florens, 2025). Such an approach can enhance habitat connectivity, maintain the flow of ecosystem services and increase resilience, which over time may prove more cost-effective (Heinen, Rahbek & Borregaard, 2020; Rafferty & Cosma, 2024).

When a non-native species negatively affects a native species but cannot be easily controlled or eradicated, it may be possible to target the mediator species or the interaction linking them, which requires a sound understanding of the indirect biotic mechanisms involved. For instance, the non-native red-whiskered bulbul (*Pycnonotus jocosus*) disperses seeds of non-native plants (Linnebjerg, Hansen & Olesen, 2009; Linnebjerg et al., 2010), indirectly harming native flora (Baider & Florens, 2011). In such cases, controlling the non-native plants may be more beneficial than attempting to control the bird itself. Controlling non-native plants will reduce availability of non-native fruits while increasing that of native fruits, plausibly shifting the bird from being problematic seed disperser to being a beneficial one, especially given the loss of many native seed dispersers due to human activities (Heinen et al., 2023).

Our review shows that although some of the non-native species may have apparent positive impacts on native species, these observations must be interpreted with caution when designing on-ground management interventions. Even though several records from this study and elsewhere are consistent (Goodenough, 2010; Vimercati et al., 2020), there is no evidence from available studies that these apparent benefits translate into improved species fitness, population recovery or range expansion. These benefits are often context-dependent, unstable over time and in many cases, represent exceptions when weighed against the overall negative impacts of non-native species (Simberloff, 2013; Bonanno, 2016; Carneiro et al., 2024). When examined contextually, in several cases, these apparent positive impacts of non-native species only occur because previous invasions have already caused habitat degradation, forcing native species to depend on non-native resources. For instance, the Telfair’s skink on Round Island consumes non-native resources where many native plants have been extirpated by overgrazing by non-native mammals (Bullock, 1977; North & Bullock, 1986). Similarly, native fruit bats increasingly rely on non-native plants for fruits (Bhanda et al., 2025) in part because non-native plant invasion reduces production of native fruits (Monty, Florens & Baider, 2013; Krivek et al., 2020) and causes native tree densities to decline (Baider & Florens, 2006). Severely invaded native habitats are used less by bats for foraging (Seegobin, Oleksy & Florens, 2024) and the bats then roost closer to commercial fruit orchards, thus visiting them more frequently during fruiting season (Seegobin, Oleksy & Florens, 2022), escalating the human-wildlife conflict. This ultimately contributed to weakening of local conservation laws (Florens, 2015; Florens, 2016) to enable mass-culling of a keystone species (Florens et al., 2017a), stressing the cascading consequences of invasion—both ecological and socio-economic. Therefore, these findings underscore that depending on non-native species because they provide sporadic and temporary benefits, can create false signals of ecosystem stability. Such benefits may delay required management actions, mask deeper ecological degradation and increase dependence on non-native species in ways that may jeopardise future conservation efforts. Active control of non-native species remains the more risk-averse and strategically sound approach, rather than leveraging their sporadic or temporary functions. This knowledge gained in Mauritius, for example, could be used to avoid future human-wildlife conflicts and to improve native forest restoration on the island of La Réunion, where *P. niger* was extinct for nearly 200 years before re-colonising it a decade or so ago (Probst & Sanchez, 2015).

Nevertheless, when backed with empirical studies, it is important for conservation managers to recognise and benefit from the positive impact brought about by non-native species. This is the case of species that are functionally similar to native species now extinct. For instance, the Aldabra tortoise (*Aldabrachelys gigantea*) and the radiated tortoise (*Astrochelys radiata*) were introduced to two offshore islets of Mauritius to reinstate ecological functions lost with the extinction of native tortoises. These include dispersing seeds of native plants, which indirectly support reptiles through the provision of food and habitat, and browsing on non-native plants, thereby reducing competitive pressure on native vegetation (Griffiths et al., 2011; Griffiths et al., 2013; Moorhouse-Gann et al., 2022). Similarly, when native species are already threatened but empirical evidence demonstrates that a non-native species increases their fitness, control of the non-native species should be carefully planned, including a phased or strategic removal, ideally coupled with the population increase or reintroduction of native analogues that have declined or gone locally extinct. Conservation managers and policymakers should employ an integrated approach taking into consideration and assessing the possible positive impacts of non-native species throughout when taking actions such as eradication or control (Kumschick et al., 2015; Kopf et al., 2017). Ignoring impacts (both positive and negative) risks causing unintended ecological impacts, such as disrupting species interactions and triggering cascading effects that ultimately threaten native species (Zavaleta, Hobbs & Mooney, 2001; Courchamp, Chapuis & Pascal, 2003; Kopf et al., 2017; Geary et al., 2019).

This review also highlights that non-native plants, and to a substantially lesser extent non-native mammals, are the most frequently reported groups with the most severe negative impacts on native species (Lorence & Sussman, 1986; Baider & Florens, 2006; Baider & Florens, 2011; Kaiser-Bunbury, Memmott & Müller, 2009; Monty, Florens & Baider, 2013; Florens et al., 2017b; Krivek et al., 2020). In Mauritius, it is therefore essential for conservation managers to prioritise the clearing of non-native plants that are highly invasive, like strawberry guava (*Psidium cattleyanum*), which is by far the most aggressive invader, forming dense stands in most of the remaining native forests (Florens et al., 2016; Florens et al., 2017b). This should be followed with controlling the most damaging non-native mammals, like rats and macaques, which harm native species including birds (Carter & Bright, 2002), plants (Bissessur et al., 2019), molluscs (Florens, Daby & Jones, 1998), and mammals (Reinegger et al., 2021). Therefore, prioritising large-scale non-native plants’ clearing followed by the control of non-native mammals would improve habitat quality at a whole-ecosystem scale, changing the current decline of most native species into recovery and reinstating or reinforcing the mutualistic interactions between them.

### Limitations of this review

To the best of our knowledge, this review offers the first comprehensive synthesis of the reported impacts of non-native species on native terrestrial biodiversity in Mauritius. The study adopts an island-wide perspective, using Mauritius as a case study that may inform similar assessments on other oceanic islands. However, this review has some limitations. Firstly, since the literature search focused only on primary empirical studies, studies lacking original data were not included. Secondly, our finding that most recorded impacts were negative may partially reflect publication and research biases, as positive and neutral effects are generally less studied and less frequently published. As a result, the predominance of negative impacts in our dataset should be interpreted cautiously, as the true impact of non-native species may be broader than what is represented in the published literature (Bayliss & Beyer, 2015). Thirdly, it is also important to note that taxonomic groups are represented at different hierarchical levels (*e.g.*, plants *vs.* mammals) and that reporting effort varies greatly among groups. Consequently, comparisons of impact frequency across taxa should be interpreted with caution, as they may partly reflect differences in research attention rather than true differences in ecological impact. Lastly, although the EICAT/EICAT+ framework provides a standard method for classifying impacts, impact records with limited information in the primary studies were conservatively placed into broader categories to maintain classification consistency. Finally, the review covers studies published up to 2023, so future reviews should include newer evidence as it becomes available.

### Knowledge gaps and future research

The review revealed important knowledge gaps requiring empirical studies. Impact records were dominated by non-native mammals and plants, and this trend was mirrored among the affected native taxa, where impacts were mostly reported for plants, reptiles, birds, and mammals. Less conspicuous native taxa, such as insects, molluscs, lichens, fungi, and bryophytes, remain overlooked, despite clear evidence that non-native species can impact them negatively (Motala et al., 2007; Baxter-Gilbert et al., 2021; Baider & Florens, 2022). This pattern likely reflects a broader trend in ecological research, in which certain groups, particularly vertebrates and plants, receive disproportionate attention simply because they are more visible, charismatic, or historically better studied (Clark & May, 2002; Kumschick et al., 2015; Velasco et al., 2015; Donaldson et al., 2017; Di Marco et al., 2017; Troudet et al., 2017; Montalvo-Mancheno et al., 2020; Llorente-Culebras, Ladle & Santos, 2023; Caldwell et al., 2024). Hence, a more equitable distribution of research effort across taxonomic groups is needed for comprehensive biodiversity conservation and to prevent the “silent extinction” of neglected taxa that may decline unnoticed (Howard & Bickford, 2014; Gerlach, Florens & Griffiths, 2025).

Most studies have focused on how non-native species affect the fitness of native individuals or populations, but far fewer have investigated their impact on biotic interactions. Within this limited research, there is a strong bias toward pollination, leaving other key biotic interactions, such as seed dispersal, frugivory, herbivory, epiphytism and predator–prey relationships underexplored. This highlights a significant knowledge gap, since ecosystem functioning depends on biotic interactions. Focusing solely on impacts at the level of biological organisation overlooks how non-native species can disrupt processes vital to ecosystem health. Understanding how non-native species modify biotic interactions is crucial for predicting long-term ecological changes and developing effective conservation management strategies. Impact mechanisms like hybridisation, parasitism, and chemical alterations of ecosystems through which non-native species may affect native biodiversity also remain neglected research areas in Mauritius. There are no published primary studies that have empirically assessed the impacts of mammals like cats, deer or feral pigs, even though these species occur in Mauritian forest and are well documented to exert significant impacts elsewhere (Dueñas et al., 2018).

Furthermore, although positive impacts of non-native species have been reported, evidence that these effects translate into longer-term fitness benefits for native species of conservation concern remains limited. This represents an important gap in the current literature and highlights the need for targeted, long-term studies designed to test whether reported positive impacts result in meaningful outcomes across relevant levels of biological organisation.

## Conclusion

This review provides the first island-wide synthesis of the ecological impacts of non-native species on Mauritius’s terrestrial biodiversity. It showed that native taxa were affected through a wide range of mechanisms, with direct mechanisms far more commonly reported than indirect ones, and the literature was strongly dominated by negative impacts. Most studies assessed impacts of non-native species at the level of biological organisation, while comparatively few examined how they alter biotic interactions. The review highlights that indirect mechanisms, although often neglected, can offer critical insights for strengthening management decisions and should therefore be considered alongside direct mechanisms when designing conservation strategies, to avoid cascading impacts across trophic levels and to improve conservation outcomes. The limited attention given to the impact of non-native species on biotic interactions may be restricting the ability to detect functional declines and emerging cases of functional extinction thus hindering the integration of interaction-based management. The findings of the review also suggest that conservation managers and policymakers could maximise benefits to native biodiversity by prioritising the removal of invasive non-native plants, particularly species like *P. cattleyanum* and subsequently the control of invasive non-native mammals. Hence, it is essential that the specific gaps in studies about the impacts of non-native species on native biodiversity highlighted here be addressed so that managers and decision-makers in Mauritius may increase the degree to which they incorporate evidence in conservation policies and management in order to make conservation more efficient and effective than it currently is.

##  Supplemental Information

10.7717/peerj.20839/supp-1 Supplemental Information 1 Number of publications per year and the cumulative number of publications on the impacts of non-native species in Mauritius from 1977 to 2023

10.7717/peerj.20839/supp-2 Supplemental Information 2 PRISMA checklist for scoping reviews (PRISMA-ScR)

10.7717/peerj.20839/supp-3 Supplemental Information 3 The 62 publications included in the review, with year, authors, title, citation, and journal information

10.7717/peerj.20839/supp-4 Supplemental Information 4 Area (km^2^), number of publications, and publication density (per km^2^) for Mauritius (remaining native habitats only) and its offshore islets

10.7717/peerj.20839/supp-5 Supplemental Information 5 Complete dataset of the 273 impact records identified in this scoping review, including non-native-native species interactions, taxonomic groups, impact mechanisms, direction of impact and study type

10.7717/peerj.20839/supp-6 Supplemental Information 6 List of distinct non-native and native species recorded in this review, grouped by status (non-native/native) and their broad taxonomic groups (plant, insects, mollusc, amphibian, reptile, bird and mammal )

10.7717/peerj.20839/supp-7 Supplemental Information 7 Detailed classification of 273 non-native –native impact records according to impact type (individual, population, community and biotic interaction) and magnitude of impact of non-native taxa For each impact record, the following information is reported: the non-native species, the native species, their respective taxonomic groups, the mechanism of interaction (classified under EICAT/EICAT+ categories), the direction of impact (negative, positive, or neutral), the impact type (individual, population, community, biotic interaction), the impact magnitude of the non-native taxa and a short explanation of the recorded impact.

## Additional Information and Declarations

### Data Availability

The following information was supplied regarding data availability: The raw data are available in the Supplemental Files and at Zenodo: https://doi.org/10.5281/zenodo.18081992
The R scripts are available at Zenodo: Seeburrun, G. (2025). R script for data processing and visualisation. Zenodo. https://doi.org/10.5281/zenodo.17798117.
