# Supplementary figures and images for: A scoping literature review on the impacts of non-native species on the native terrestrial biodiversity of an oceanic island
Source: PeerJ. 2026 Feb 25;14:e20839. doi: 10.7717/peerj.20839 (PMC12949587; doi:10.7717/peerj.20839)

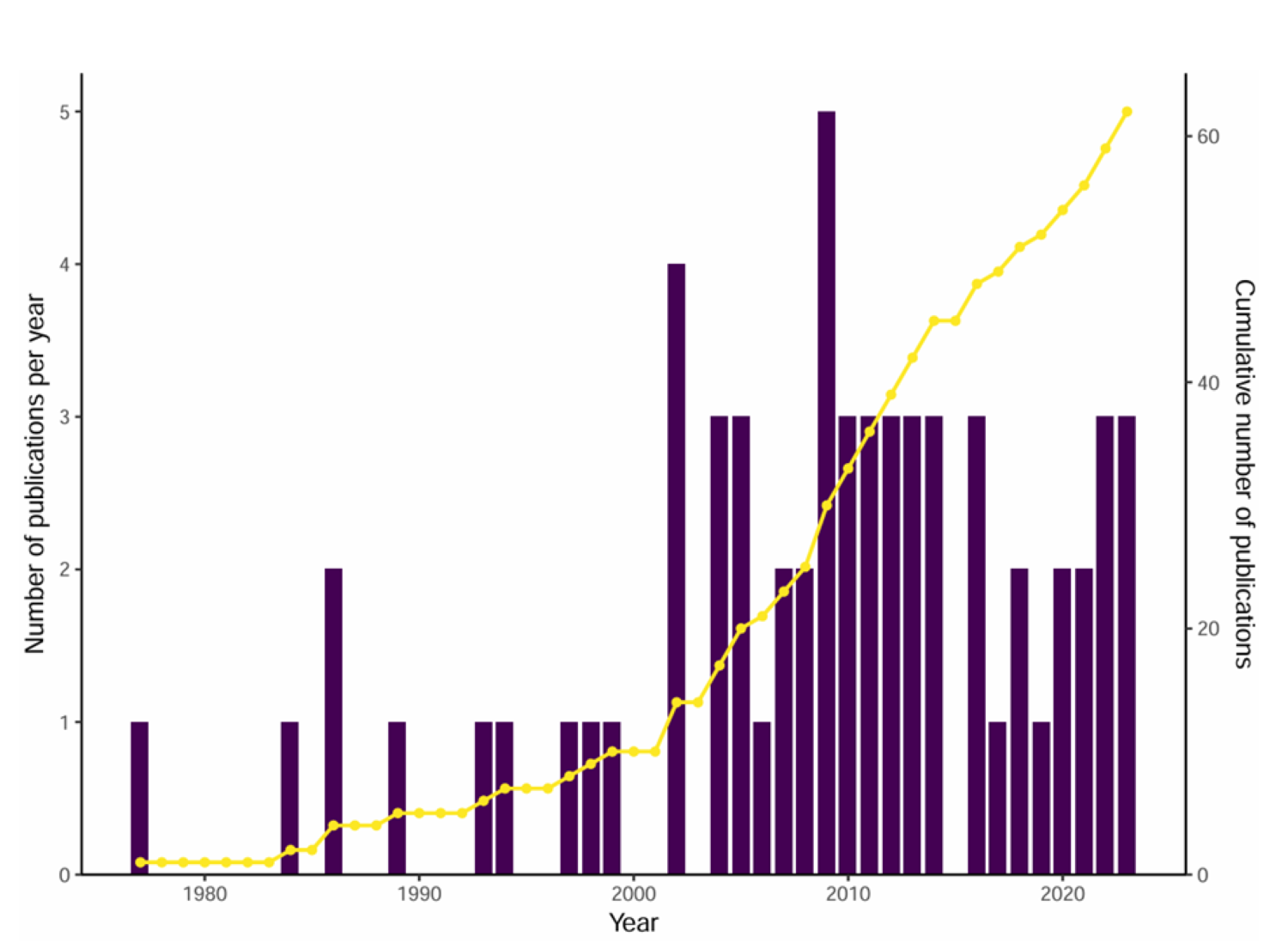

Supplement: Supplemental Information 1 [file peerj-14-20839-s001.png]
